# Effect of coagulation factor concentrate administration on ROTEM® parameters in major trauma

**DOI:** 10.1186/s13049-015-0165-4

**Published:** 2015-10-29

**Authors:** Martin Ponschab, Wolfgang Voelckel, Michaela Pavelka, Christoph J. Schlimp, Herbert Schöchl

**Affiliations:** Ludwig Boltzmann Institute for Experimental and Clinical Traumatology, AUVA Research Centre, Vienna, Austria; Department of Anaesthesiology and Intensive Care Medicine, AUVA Trauma Centre Salzburg, Academic Teaching Hospital of the Paracelsus Medical University, Franz Rehrl Platz 5, 5020 Salzburg, Austria

**Keywords:** Fibrinogen concentrate, Prothrombin complex concentrates, ROTEM, Trauma

## Abstract

**Background:**

Purified coagulation factor concentrates, such as fibrinogen concentrate (FC) and prothrombin complex concentrate (PCC) are increasingly used as haemostatic therapy for trauma-induced coagulopathy (TIC). The impact of FC and PCC administration on ROTEM parameters among patients with TIC has not been adequately investigated.

**Methods:**

In this retrospective observational study, changes to ROTEM parameters, induced by three different therapeutic interventions, were investigated: patients receiving FC only (FC-group); patients treated with FC and PCC (FC + PCC-group) and patients treated with PCC only (PCC-group).

**Results:**

The study population comprised 96 patients who were predominately male (69 [71.9 %]), median age was 45.0 (26.3–60.0) years, and the median injury severity score was 34.0 (25.0–44.5). Administration of FC resulted in a significant reduction of the clotting time (CT) in both the EXTEM and FIBTEM assays but had no effect on INTEM CT. Clot amplitude (CA) increased significantly in the FIBTEM assay but remained unchanged in the EXTEM and INTEM assays. The combined administration of FC and PCC increased FIBTEM maximum clot firmness (MCF) and normalized EXTEM CT but did not change either INTEM or FIBTEM CT. Following PCC therapy, EXTEM and FIBTEM CT normalized; CA at 10 min after CT measurements decreased significantly in EXTEM, INTEM and FIBTEM.

**Conclusions:**

Administration of FC alone or in combination with PCC resulted in a significant improvement of fibrin polymerisation as measured by an increase in FIBTEM MCF. CT is dependent not only on thrombin generation but also on the availability of substrate (fibrinogen). Accelerated fibrin polymerisation rate results in earlier clot formation and consequently shorter CT. PCC administration normalised EXTEM CT below the upper threshold of 80 s.

This study was performed at the AUVA Trauma Centre Salzburg, Salzburg, Austria.

**Electronic supplementary material:**

The online version of this article (doi:10.1186/s13049-015-0165-4) contains supplementary material, which is available to authorized users.

## Background

Trauma induced coagulopathy (TIC), which is accompanied by diffuse uncontrollable microvascular bleeding, is still associated with high mortality rate [1]. According to standard coagulation tests (SCTs), 24–34 % of trauma patients are coagulopathic at emergency room (ER) admission [2, 3]. This early trauma-related coagulopathy is associated with a high risk for massive transfusion and unfavourable outcome [4].

The implementation of a number of treatment strategies, such as permissive hypotension, restrictive fluid administration and aggressive temperature control has proven efficient to reduce trauma-related exsanguination [5]. Furthermore, early and aggressive haemostatic therapy is a cornerstone of modern bleeding management and has been shown to improve survival in coagulopathic trauma patients [6, 7]. In most trauma facilities worldwide, fresh frozen plasma (FFP) is the haemostatic agent used to restore lacking coagulation factors [5, 8, 9]. Due to logistical reasons, FFP transfusion is often associated with substantial time delays and only busy trauma units store pre-thawed plasma [10]. Moreover, whole blood reconstituted from the three components, red blood cells (RBCs), FFP and platelet concentrate (PC), contains substantially lower amounts of coagulation factors, in particular fibrinogen, compared with native whole blood [11]. Furthermore, the ideal ratio of FFP to RBCs is still a matter of debate [10].

An alternative approach for rapid replenishment of lacking coagulation factors is the use of purified coagulation factor concentrates, such as fibrinogen concentrate (FC) or prothrombin complex concentrate (PCC) [12–16]. In contrast to FFP, these haemostatic agents contain a well-defined concentration of coagulation proteins, they are immediately available and blood group matching is not necessary [12].

Viscoelastic tests (VETs), most commonly thrombelastography (TEG®, Haemoscope-Haemonetics, Niles, IL) and thromboelastometry (TEM®, Tem Systems Inc., Durham, NC), provide a valuable alternative, or an adjunct, to SCTs in the setting of bleeding, and are increasingly used to diagnose coagulation disturbances following trauma [8, 17–19]. VETs provide a comprehensive overview of the whole coagulation process and are assumed to be superior to prothrombin time (PT) or activated thromboplastin time (aPTT) [20–22]. Furthermore, VETs can be run as a point of care haemostatic monitoring device, which has been shown to provide measurements much quicker than SCTs [23].

Although some trauma units, in particular in Central Europe, use VET results to guide coagulation therapy based on coagulation factor concentrates, little is known about the extent to which these haemostatic agents change viscoelastic parameters in major bleeding trauma patients. Therefore, we investigated ROTEM® findings before and after administration of FC, PCC or a combination of both.

## Methods

Following local ethics committee approval (415-EP/73/197-2013) we performed a retrospective analysis of major trauma patients admitted to the AUVA Trauma Centre, Salzburg between 2008 and 2015. The inclusion criteria were treatment of coagulopathy with coagulation factor concentrates and valid ROTEM results measured before and after administration of coagulation factor concentrates. The maximum time interval between the two measurements was 80 min; sets of analyses were excluded if VET results following therapeutic intervention were obtained later than 80 min after the initial ROTEM analysis. This time frame was chosen as it takes approximately 15 min to obtain the first test results from the ROTEM device. Following this, preparation of the factor concentrates takes approximately 10–15 min. According to the manufacturer’s recommendation, FC should be completely dissolved after a maximum time of 15 min under light circling and avoiding heavy shaking which can cause foam generation. In our institution, FC and sterile water for dissolution is stored at room temperature in the emergency room as low temperature of the carrier fluid can prolong the process of dissolution. The manufacturer recommends that 1 g FC dissolved in 50 ml should be applied with a maximum speed of 5 ml/min or within a minimum of 10 min under normal conditions. However, in severe bleeding FC is administered much faster. In less severe cases, we infuse FC by motor pump with a high administration rate (4 g within 15 min). Infusion rates up to 6 g in 1–2 min have been reported in the literature [24]. PCC is administered intravenously by hand from the syringe connected to the venous access. According to the manufacturer, a maximum infusion rate of 210 U/min should not be exceeded. Thus, it takes approximately 2–3 min for application. Taken together administration of coagulation factor concentrates takes another 10 – 15 min.

### Coagulation therapy (algorithm-based)

Coagulation management was based on our institutional protocol for bleeding trauma patients, with treatment determined by the results of VETs (ROTEM®, Tem International, Munich, Germany) [12] Briefly, ROTEM analyses are performed upon emergency room (ER) admission or during initial operative treatment, and if a patient is still coagulopathic during intensive care unit (ICU) stay. FC (Haemocomplettan P, CSL Behring, Marburg, Germany) is considered as first-line therapy in patients with impaired fibrin polymerisation, i.e., a clot amplitude of <7 mm at 10 minutes after CT measurement in the FIBTEM assay (target FIBTEM CA10: 10–12 mm). If clotting time (CT) in the extrinsically activated assay (EXTEM) remains prolonged (>80 s) following FC treatment, PCC is administered. For patients with obviously severe coagulopathy and life-threatening bleeding, immediate treatment with both FC and PCC is preferred. Tranexamic acid (TXA; 15–20 mg/kg bodyweight for patients with injury severity score [ISS] ≥16 and/or severe shock) was incorporated into our treatment protocol in 2011 following publication of the CRASH-2 data [25]. In all cases, the attending physician was free to treat patients at his/her own discretion.

ROTEM measurements were performed before and after three different therapeutic interventions: FC only (FC-group); FC in combination with PCC (FC + PCC-group); and PCC only (PCC-group). Demographic data, laboratory data, trauma severity scores, including ISS, new ISS (NISS), Glasgow coma scale (GCS) score and outcomes data were obtained from the electronic trauma database.

### Standard coagulation tests

Citrated blood samples were centrifuged and the following coagulation parameters were assessed: fibrinogen concentration (Clauss Method with electro-mechanical detection using a STA-Compact analyser [Diagnostica Stago S.A.S., Asnières sur Seine, France]; normal range 200–450 mg/dL), PT (measured using a Sysmex XE-2100 [Roche Diagnostics, Mannheim, Germany]; normal range 11.0–16.1 s), aPTT (measured using Sysmex XE-2100; [Roche Diagnostics, Mannheim, Germany]; normal range 23.7–34.9 s) and antithrombin III (AT III; normal range 80–120 %). Haemoglobin and platelet count were determined on a Sysmex SF-3000 (Sysmex Corporation, Kobe, Japan) and base excess was determined on a Roche OMNI® S Blood Gas Analyser (Roche Diagnostics, Mannheim, Germany).

### Statistical analysis

For all parameters, normality of the data distribution was tested using the Kolmogorov-Smirnov test. The results are expressed as mean ± standard deviation or median (interquartile range; 25th percentile–75th percentile). Between-group differences were analysed using a paired *t*-test or Wilcoxon matched-pairs signed-rank test as appropriate. Statistical calculations were performed using GraphPad Prism 5.03 (GraphPad Software, La Jolla, CA). The level of significance was set at *P* < 0.05.

## Results

The study population comprised 96 patients who were predominately male (69; 71.9 %) with a median age of 45.0 (26.3–60.0) years. The median injury severity score (ISS) was 34.0 (25.0–44.5), the median NISS was 41.0 (27.0–50.0), and the median Glasgow coma score (GCS) was 10.0 (5.0–15.0). Demographic and clinical data at ER admission are depicted in Table 1.

**Table 1:** Clinical characteristics of patients at emergency room admission

	All patients
Number of patients, n	96
Age (years)	45 (26.3–60.0)
Male, n (%)	69 (71.9)
Weight (kg)	80 (73–90)
GCS	10.0 (5–15.0)
ISS	34 (25–44.5)
NISS	41 (27–50)
Systolic BP (mm Hg)	101.9 ± 34.2
Pulse (min^−1^)	96.2 ± 30.7
Temperature (°C)	35.85 (35.0–36.25)

Values are presented as n (%), mean ± standard deviation or median (interquartile range)

*BP* blood pressure, *GCS* Glasgow coma scale, *ISS* injury severity score, *NISS* new injury severity score, *RTS* revised trauma score

One hundred and twenty-five pre-therapy/post-therapy pairs of ROTEM analyses were selected for inclusion in this study. The median time interval between the analyses within each pair was 53.0 (40.0–66.0) minutes. Following FC administration (FC-group), 89 pairs of ROTEM measurements were available for analysis. A further 23 measurements were performed after combined treatment with FC and PCC (FC + PCC-group) and 13 pairs of measurements were completed after therapy with PCC only (PCC-group). Laboratory data at ER admission are outlined in Table 2. Blood transfusion and coagulation therapy within the first 24 h is depicted in Table 3.

**Table 2:** Blood cell count, standard coagulation tests and blood gas analysis at emergency room admission

	All patients
Hb (g/dL)	10.94 ± 2.8
Platelet count (10^3^/μL)	192.5 (165.3–226.8)
PTI (%)	64.9 ± 23.3
PT (s)	16.4 (14.6–19.3)
aPTT (s)	31.8 (27.45–40.3)
Fibrinogen (g/dL)	149.0 (110.8–199.0)
pH	7.3 (7.24–7.351)
BE (mmol/L)	−4.85 (8.0–2.7)
Lactate (mmol/L)	3.1 (2.1–4.4)

Values are presented as mean ± standard deviation or median (interquartile range)

*aPTT* activated thromboplastin time, *BE* base excess, *Hb* hemoglobin, *PTI* prothrombin time index, *PT* prothrombin time

**Table 3:** Blood transfusion and coagulation therapy within the first 24 h

	All patients
RBC (units)	7 (3–10)
FFP (units)	0 (0–0)
PC (units)	0 (0–0)
PCC (units)	0 (0–1800)
FC (g)	6 (3–9)
TXA (g)	0 (0–1)

Values are presented as median (interquartile range)

*FC* fibrinogen concentrate, *FFP* fresh frozen plasma, *PC* platelet concentrate, *PCC* prothrombin complex concentrate, *RBC* red blood cells, *TXA* tranexamic acid

### ROTEM parameters before and after therapy with coagulation factor concentrates

**Fig. 1:**
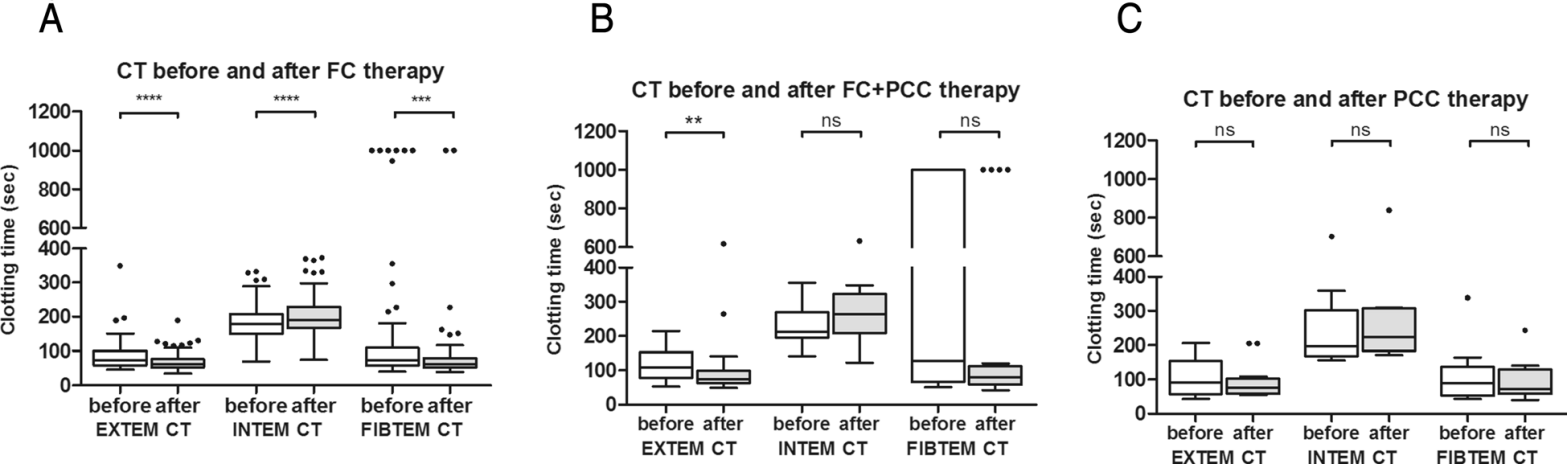
Changes in thromboelastometric clotting time before and after treatment with coagulation factor concentrates. CT, clotting time; EXTEM, extrinsically activated test; FC, fibrinogen concentrate; INTEM, intrinsically activated test; PCC, prothrombin complex concentrate. Values are presented as box and whiskers plots (Tukey), median (interquartile range). ns = not significant; **P < 0.01; ***P < 0.001; ****P < 0.0001

**Fig. 2:**
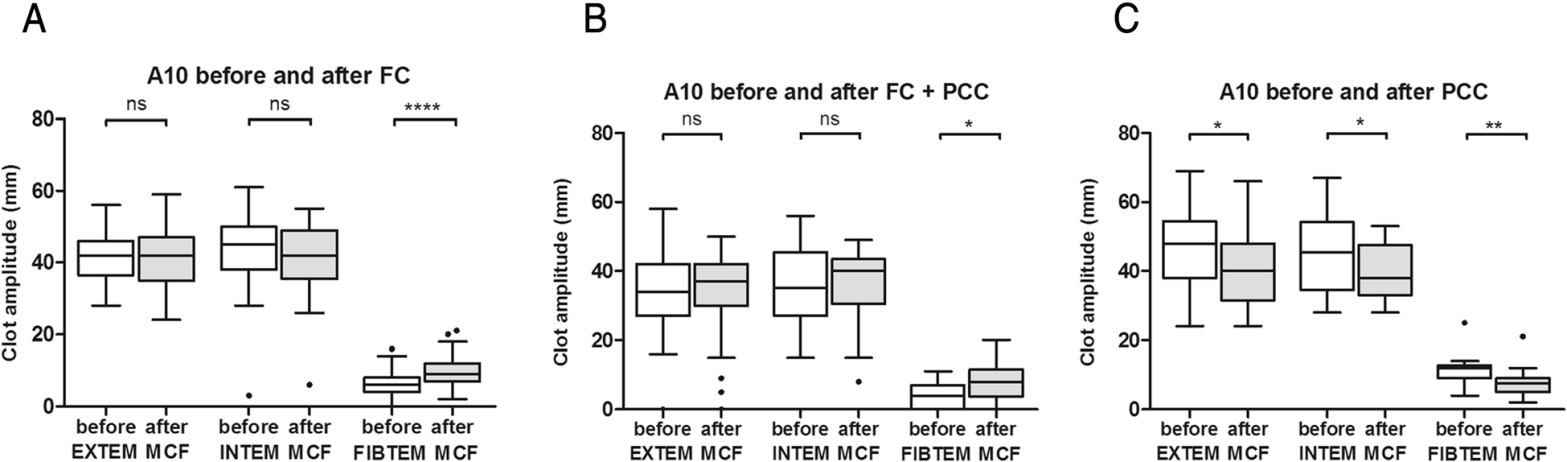
Changes in thromboelastometric clot amplitude after 10 min running time before and after treatment with coagulation factor concentrates. CA 10, clot amplitude after 10 min running time; EXTEM, extrinsically activated test; FC, fibrinogen concentrate; FIBTEM, extrinsically activated test plus cytochalasin D; INTEM, intrinsically activated test; PCC, prothrombin complex concentrate. Values are presented as box and whiskers plots (Tukey), median (interquartile range). ns = not significant; *P < 0.05; **P < 0.01; ****P < 0.0001

A median dose of 4 (3–4) g FC resulted in a significant reduction in EXTEM CT (*P* < 0.0001) and FIBTEM CT (*P* = 0.0002). In contrast, FC administration had no effect on INTEM CT, which was significantly prolonged between the two measurements (*P* < 0.0001; Fig. 1a and Additional file 1: Table S1). Following treatment with FC, a significant increase in alpha angle in both, EXTEM and INTEM assay was observed (*P* < 0.0001 and *P* = 0.024). In addition, there was a significant increase in FIBTEM clot amplitude at 10 minutes after CT measurement (CA10; *P* < 0.0001). In contrast, INTEM CA10 and EXTEM CA10 remained unchanged (Additional file 1: Table S1). Furthermore, treatment with FC resulted in a significant increase in FIBTEM maximum clot firmness (MCF) from 7 to 11 mm (*P* < 0.0001; Fig. 2a and Additional file 1: Table S1). There was no change in either EXTEM or INTEM MCF.

A combined therapy of 5.5 (4–8) g FC and 1200 (1200–1800) units PCC significantly reduced EXTEM CT only (*P* = 0.0093). In contrast, INTEM CT was prolonged following treatment (Fig. 1b). However, this did not reach statistical significance. Neither EXTEM nor INTEM MCF changed significantly. However, there was a significant, clinically relevant increase in FIBTEM MCF from 4.5 to 8 mm (*P* = 0.012; Fig. 2b and Additional file 1: Table S1).

Following administration of 1200 (600–1500) units PCC, normalization of both EXTEM CT from 101 s (73.5–171.8) to 77.0 s (64.75–122.5) and FIBTEM CT from 92.0 (56–155.5) to 78 (61.5–117.5) was observed. However, this difference did not reach statistical significance (Fig. 1c). INTEM CT was prolonged, but remained with normal limits (*P* = 0.151).

CA10 decreased significantly in EXTEM (48.0 to 40.0; *P* = 0.021), INTEM (45.5 to 38.0; *P* = 0.029) and FIBTEM CA10 (12.0 to 7.5 mm; *P* = 0.005) (Fig. 2). MCF decrease in EXTEM and FIBTEM but reached significance in INTEM only. (*P* = 0.033) (Additional file 1: Table S1).

All ROTEM parameters measured before and after treatment with coagulation factor concentrates are shown in Additional file 1: Table S1.

## Discussion

VETs are increasingly used to diagnose coagulopathy and guide haemostatic therapy in many trauma units worldwide [7, 8, 10, 26]. Moreover, coagulation factor concentrates are part of massive transfusion protocols in many European trauma facilities in order to rapidly improve haemostatic capacity in severely bleeding patients [16, 27, 28]. To the best of our knowledge, this is the first study to investigate ROTEM® parameters following administration of coagulation factor concentrates in a trauma population.

As expected FC administration increased both FIBTEM CA10 and MCF in most of the patients studied. In contrast, the detected increases in FIBTEM CA10 and MCF were not followed by significant increases in INTEM and EXTEM CA10 or MCF. Clot strength is dependent on the interaction of activated platelets, the build-up of the fibrin network and activated factor XIII [29]. An explanation for this finding is that a lack of one of these components responsible for clot firmness can potentially be compensated for by another one. In major shock and blood loss both fibrinogen and platelet count decrease, resulting in diminished clot amplitude. An increase in the fibrin component of the clot might counterbalance the decrease in platelet count. In the current study, administration of FC resulted in an increase in FIBTEM CA10 and MCF whereas EXTEM and INTEM CA10 and MCF remained unchanged. The reduction in the EXTEM CA after FC administration is in our opinion a result of ongoing blood loss and consumption. Platelets are the predominate component of the EXTEM CA. If FIBTEM CA increases following FC administration and EXTEM CA remains more or less unchanged, this finding is highly suggestive of diminished platelet count and/or function. Moreover, this finding is a strong hint that FC administration might have a compensatory effect on clot amplitude if platelet count is low. This observation is in agreement with experimental studies proposing a compensatory role of fibrinogen on clot firmness in animals with low platelet count. Velik-Salchner et al. studied the effect of FC or PC administration on blood loss in a porcine model of thrombocytopenia [30]. FC administration in thrombocytopenic pigs resulted in a significantly better increase in median clot firmness than following platelet transfusion (*P* = 0.0004). Interestingly, thrombocytopenic animals treated with FC showed lower cumulative blood loss and higher survival rate compared with pigs treated with PC only [30].

Moreover, FC infusion not only increased clot firmness but also diminished clot lysis. Improved fibrin polymerization following FC application established clots with higher resistance to profibrinolytic stimuli. This finding is in agreement with data from the MATTERs 2 study, which revealed that inhibition of lysis by TXA resulted in an equal improvement in outcome than transfusion of cryoprecipitate alone. The combination of both substances exhibited the best effect on survival [31].

Importantly, in the current study EXTEM CT shortened significantly following FC administration. By definition, CT is the time from initiation of the coagulation process until a clot amplitude of 2 mm is reached [29]. This finding suggests that if a higher quantity of substrate (e.g., fibrinogen) is available for initial clot formation, EXTEM clot amplitude of 2 mm will be reached more quickly, and consequently CT will be shorter. These results are in line with findings reported by Bolliger et al., who used thromboelastometry to evaluate speed and quality of clot formation in diluted samples supplemented with 50–300 mg/dL fibrinogen. The addition of increasing amounts of fibrinogen led to a concentration-dependent improvement in all thromboelastometric parameters including EXTEM CT [32]. This was also observed by our group in a porcine animal model with in vivo haemodilution and intravenous or intraosseous administration of FC. After administration of FC, EXTEM MCF increased and EXTEM CT was significantly shortened, despite not having added a coagulation factor that enhances thrombin generation [33].

Interestingly, this finding was not detected with INTEM CT. There are several possible explanation for this observation. The endothelial glycocalyx contains significant amounts of heparin-like material [34, 35]. Glycocalyx shedding, as a consequence of shock and hypoperfusion, might discharge these substances resulting in relevant amounts of heparin in the blood stream [36, 37]. A prospective observational study including 77 major trauma patients demonstrated that 5 % of the study cohort displayed evidence of auto-heparinisation [38]. In contrast to EXTEM, the INTEM assay is heparin sensitive [29]. Therefore, the observed prolongation of INTEM CT between the two measurements might be a consequence of heparin release. Furthermore, some PCC preparations contain significant amounts of heparin, which may be a possible explanation for the observed prolongation in INTEM CT following PCC administration. Scharbert et al. reported a concentration-dependent prolongation in CT and clot formation time in non-activated ROTEM tests (NATEM) with a heparin PCC preparation compared with a heparin-free PCC, even at the lowest concentration [39]. In contrast to our study, these experiments were conducted in vitro, without any influence of metabolism of heparin by the liver. However, in the current study we also observed a prolongation in INTEM CT in the FC-group, which did not receive any PCC. A further reason for the observed prolongation of INTEM CT could be related to low factor XII concentrations. Factor XII deficiency is not associated with bleeding diathesis, but might prolong coagulation tests related to contact activation [40].

Administration of PCC alone was associated with reductions in CA in both EXTEM and FIBTEM but a shortening of the CT. PCC primarily augments thrombin generation; as such, it has only limited effects on clot firmness. The detected decrease in CA observed in the current study is presumably a result of ongoing blood loss between the measurements, with consecutive loss of platelets and fibrinogen. Thus, both EXTEM CA and FIBTEM CA decreased but EXTEM CT shortened due to augmented thrombin generation.

### Limitations

Several limitations of this study have to be acknowledged. The analyses were not restricted to three groups of patients but to different treatment regimens. This means that the same patient could have received FC and at a later time a combination of FC and PCC or PCC alone, which is in accordance with our goal directed haemostatic algorithm [12]. Therefore, patients were not stratified to an individual treatment group.

As this is a retrospective study, detailed information of intraoperative blood loss, fluid therapy and RBC transfusion between the two measurements was not available. The mean time interval between the two measurements was less than one hour. However, in severely bleeding patients high amounts of fluids and RBCs may have been transfused, which might affect the results. Moreover, artificial colloids interfere with fibrin polymerisation. In our institution, gelatins are the main artificial colloids used. In contrast to hydroxyethyl starches, fibrin polymerization is less disturbed by gelatins and can be compensated for more easily by FC administration [41]. Those patients who received both FC and PCC were considered more coagulopathic than patients who were treated with one component only. Therefore, observed changes between the groups are not comparable. However, it does not necessarily mean that the coagulation factor therapy was less efficient.

## Conclusions

The current study showed that administration of FC caused higher amounts of substrate to be available for initial clot formation, resulting in a shortening of the CT in both the EXTEM and FIBTEM assays. Clot amplitude improved in FIBTEM only and remained unchanged in EXTEM and INTEM, which could be attributed to a decrease in concomitant platelet count between the measurements. Combined therapy of FC and PCC resulted in a significant improvement in fibrin polymerization without substantial changes in EXTEM and INTEM clot amplitude. INTEM CT never improved, which might be a result of shock related “auto-heparinisation” or Factor XII deficiency. PCC administration normalised EXTEM CT below the upper threshold of 80 s.

## Additional file

Additional file 1: Table S1. ROTEM parameters measured before and after treatment with coagulation factor concentrates. (DOCX 28 kb)

## Abbreviations

aPTT: Activated partial thromboplastin time
AT III: Antithrombin III
CA10: Clot amplitude after 10 min
CT: Clotting time
ER: Emergency room
FC: Fibrinogen concentrate
FFP: Fresh frozen plasma
GCS: Glasgow coma scale
ICU: Intensive care unit
ISS: Injury severity score
MCF: Maximum clot firmness
NISS: New injury severity score
PC: Platelet concentrate
PCC: Prothrombin complex concentrate
PT: Prothrombin time
PTI: Prothrombin time index
RBC: Red blood cell
SCT: Standard coagulation test
TXA: Tranexamic acid
VET: Viscoelastic test
