# Supplementary material for: Effect of coagulation factor concentrate administration on ROTEM® parameters in major trauma
Source: Scand J Trauma Resusc Emerg Med. 2015 Oct 29;23:84. doi: 10.1186/s13049-015-0165-4 (PMC4625604; doi:10.1186/s13049-015-0165-4)
Supplement: Additional file 1: Table S1. — ROTEM parameters measured before and after treatment with coagulation factor concentrates. (DOCX 28 kb) [file 13049_2015_165_MOESM1_ESM.docx]

**Additional file 1**

**Table S1: ROTEM parameters measured before and after treatment with coagulation factor concentrates**

| \|  \|  \|  \|  \|  \| \| \| \|  \|  \| \| \| \| \| \|  \|  \| \| \| \| --- \| --- \| --- \| --- \| --- \| --- \| --- \| --- \| --- \| --- \| --- \| --- \| --- \| --- \| --- \| --- \| --- \| --- \| --- \| \|  \|  \|  \|  \|  \| **FC only 4 (3–4) g** \|  \| **FC 5.5 (4–8) g + PCC 1200 (1200–1800) U** \| \| \| \| \| \|  \| **PCC only 1200 (600–1500) U** \| \| \| \| \| \|  \|  \| **EXTEM** \| **n** \| Before FC \| After FC \| p-value \| **n** \| Before FC and PCC \| \| \| After FC and PCC \| p-value \| n \| Before PCC \| \| \| After PCC \| p-value \| \|  \|  \| CT (sec) \| 89 \| 73 (58.5–101) \| 62 (53–77) \| **<0.0001** \| 23 \| 108 (79–153) \| \| \| 75 (62–99) \| **0.0093** \| 13 \| 92 (57–154.5) \| \| \| 76 (58.5–102) \| 0.310 \| \|  \|  \| A10 (mm) \| 89 \| 42 (36.5–46) \| 42 (35–47) \| 0.709 \| 23 \| 34 (27–42) \| \| \| 37 (30–42) \| 0.758 \| 13 \| 48 (38–54.5) \| \| \| 40 (31.5–48) \| **0.021** \| \|  \|  \| MCF (mm) \| 89 \| 52 (48–56) \| 54 (49–58) \| 0.280 \| 23 \| 47 (34.5–55.25) \| \| \| 48.5 (41.25–55.75) \| 0.603 \| 13 \| 59 (48.5–62.5) \| \| \| 48 (44.5–58.5) \| 0.062 \| \|  \|  \| CFT (sec) \| 89 \| 166 (135.5–206) \| 149 (121.5–205.5) \| 0.431 \| 19 \| 239.5 (133.5–370) \| \| \| 166.5 (109.8–235) \| **0.031** \| 13 \| 118 (99–167) \| \| \| 145 (130.5–242) \| **0.005** \| \|  \|  \| Alpha (°) \| 87 \| 59 (55–66) \| 68 (60–73) \| **<0.0001** \| 20 \| 56 (51–66.75) \| \| \| 68 (57–75) \| 0.218 \| 13 \| 72.5 (68.5–76.5) \| \| \| 60.5 (69.75) \| **0.011** \| \|  \|  \| ML (%) \| 85 \| 3 (0.5–6.5) \| 5 (1–8) \| 0.197 \| 22 \| 3.5 (0–62.5) \| \| \| 1 (0–9) \| 0.245 \| 13 \| 1 (0–6) \| \| \| 3 (1–6.5) \| 0.894 \| \|  \|  \|  \|  \|  \|  \|  \|  \|  \| \| \|  \|  \|  \|  \| \| \|  \|  \| \|  \|  \| **INTEM** \|  \|  \|  \|  \|  \|  \| \|  \| \|  \|  \|  \| \| \|  \|  \| \|  \|  \| CT (sec) \| 69 \| 178 (149–207.5) \| 191 (167–229) \| **<0.0001** \| 17 \| 212 (195–269) \| \| 264 (207.5–322) \| \| 0.233 \| 12 \| 197 (168–301.5) \| \| \| 224.5(183.5–307.5) \| 0.151 \| \|  \|  \| A10 (mm) \| 69 \| 45 (38–50) \| 42 (35.5–49) \| **0.036** \| 17 \| 35 (27–45.5) \| \| 40 (30.5–43.5) \| \| 0.854 \| 12 \| 45.5 (34.5–54.25) \| \| \| 38 (33–47.5) \| **0.029** \| \|  \|  \| MCF (mm) \| 69 \| 53 (48–58) \| 53 (48–57) \| 0.747 \| 17 \| 45 (37–54) \| \| 49 (39.5–55) \| \| 0.953 \| 12 \| 55 (46–62.25) \| \| \| 48 (44.25–54.5) \| **0.033** \| \|  \|  \| CFT (sec) \| 68 \| 121.5 (98–164.8) \| 127 (97.25–187.5) \| 0.466 \| 13 \| 202 (98–239) \| \| 137(103–217) \| \| 0.296 \| 12 \| 118.5 (78.5-215.8) \| \| \| 156 (108.5–230.3) \| 0.074 \| \|  \|  \| Alpha (°) \| 67 \| 68 (63–72) \| 71 (64–74) \| **0.024** \| 13 \| 64 (57.25–72) \| \| 70 (50.5–73) \| \| 0.424 \| 12 \| 71 (67–74) \| \| \| 65 (56–71) \| 0.072 \| \|  \|  \| ML (%) \| 64 \| 2 (0–5) \| 3.5 (1–8) \| 0.082 \| 16 \| 1.0 (0–10.25) \| \| 0.5 (0–5.25) \| \| 0.385 \| 12 \| 1 (0–5.75) \| \| \| 1.5 (0–5.75) \| 0.905 \| \|  \|  \|  \|  \|  \|  \|  \|  \|  \| \|  \| \|  \|  \|  \| \| \|  \|  \| \|  \|  \| **FIBTEM** \| \|  \|  \|  \|  \|  \| \|  \| \|  \|  \|  \| \| \|  \|  \| \|  \|  \| CT (sec) \| 88 \| 72.5 (57–108.8) \| 60.5 (53–80.5) \| **0.0002** \| 22 \| 127 (65.75–1727) \| \| 80.5 (58–112.5) \| \| 0.126 \| 12 \| 89.5 (52–137) \| \| \| 73 (58.5–129.3) \| 0.388 \| \|  \|  \| A10 (mm) \| 88 \| 6 (4–8) \| 9 (7–12) \| **<0.0001** \| 22 \| 4 (0–7) \| \| 8 (3.75–11.5) \| \| **0.016** \| 12 \| 12 (9–12.75) \| \| \| 7.5 (5–9) \| **0.005** \| \|  \|  \| MCF (mm) \| 88 \| 7 (5–8.75) \| 10 (8–13) \| **<0.0001** \| 22 \| 4.5 (0–7.25) \| \| 8 (5.5–13) \| \| **0.012** \| 12 \| 12.5 (9.25–14) \| \| \| 8.5(5–11.75) \| 0.053 \| \|  \|  \| ML (%) \| 88 \| 6 (0.25–23) \| 2 (0–8) \| **0.0156** \| 19 \| 12 (3–100) \| \| 3 (0–12) \| \| 0.079 \| 12 \| 0 (0–3) \| \| \| 0 (0–3) \| 0.461 \| |  |  |  |  |  |  |  |  |  |  |  |  |
| --- | --- | --- | --- | --- | --- | --- | --- | --- | --- | --- | --- | --- | --- | --- | --- | --- | --- | --- | --- | --- | --- | --- | --- | --- | --- | --- | --- | --- | --- | --- | --- | --- | --- | --- | --- | --- | --- | --- | --- | --- | --- | --- | --- | --- | --- | --- | --- | --- | --- | --- | --- | --- | --- | --- | --- | --- | --- | --- | --- | --- | --- | --- | --- | --- | --- | --- | --- | --- | --- | --- | --- | --- | --- | --- | --- | --- | --- | --- | --- | --- | --- | --- | --- | --- | --- | --- | --- | --- | --- | --- | --- | --- | --- | --- | --- | --- | --- | --- | --- | --- | --- | --- | --- | --- | --- | --- | --- | --- | --- | --- | --- | --- | --- | --- | --- | --- | --- | --- | --- | --- | --- | --- | --- | --- | --- | --- | --- | --- | --- | --- | --- | --- | --- | --- | --- | --- | --- | --- | --- | --- | --- | --- | --- | --- | --- | --- | --- | --- | --- | --- | --- | --- | --- | --- | --- | --- | --- | --- | --- | --- | --- | --- | --- | --- | --- | --- | --- | --- | --- | --- | --- | --- | --- | --- | --- | --- | --- | --- | --- | --- | --- | --- | --- | --- | --- | --- | --- | --- | --- | --- | --- | --- | --- | --- | --- | --- | --- | --- | --- | --- | --- | --- | --- | --- | --- | --- | --- | --- | --- | --- | --- | --- | --- | --- | --- | --- | --- | --- | --- | --- | --- | --- | --- | --- | --- | --- | --- | --- | --- | --- | --- | --- | --- | --- | --- | --- | --- | --- | --- | --- | --- | --- | --- | --- | --- | --- | --- | --- | --- | --- | --- | --- | --- | --- | --- | --- | --- | --- | --- | --- | --- | --- | --- | --- | --- | --- | --- | --- | --- | --- | --- | --- | --- | --- | --- | --- | --- | --- | --- | --- | --- | --- | --- | --- | --- | --- | --- | --- | --- | --- | --- | --- | --- | --- | --- | --- | --- | --- | --- | --- | --- | --- | --- | --- | --- | --- | --- | --- | --- | --- | --- | --- | --- | --- | --- | --- | --- | --- | --- | --- | --- | --- | --- | --- | --- | --- | --- | --- | --- | --- | --- | --- | --- | --- | --- | --- | --- | --- | --- | --- | --- | --- | --- | --- | --- | --- | --- | --- | --- | --- | --- | --- | --- | --- | --- | --- | --- | --- | --- | --- | --- | --- | --- | --- | --- | --- | --- | --- | --- | --- | --- | --- | --- | --- | --- | --- | --- | --- | --- | --- | --- | --- | --- | --- | --- | --- | --- | --- | --- | --- | --- | --- | --- | --- | --- | --- | --- | --- | --- | --- | --- | --- | --- | --- | --- | --- | --- | --- | --- | --- | --- | --- | --- | --- | --- | --- | --- | --- | --- | --- | --- | --- | --- | --- | --- | --- | --- | --- | --- | --- | --- | --- | --- | --- | --- | --- | --- | --- | --- | --- | --- | --- | --- | --- | --- | --- | --- | --- | --- |
|  |  |  |  |  |  |  |  |  |  |  |  |  |
| CT, clotting time; CFT, clot formation time; A10, clot amplitude after 10 min; MCF, maximum clot firmness; alpha; alpha angle; ML, maximum lysis;  Values are presented as mean and standard deviation or median [25^th^ percentile - 75^th^ percentile]. |  |  |  |  |  |  |  |  |  |  |  |  |
